# 胸壁缺损的修复与重建

**DOI:** 10.3779/j.issn.1009-3419.2018.04.08

**Published:** 2018-04-20

**Authors:** 高明 肖

**Affiliations:** 湖南省肿瘤医院 Hunan Provincial Tumor Hospital

肿瘤切除或外伤等原因导致胸壁缺损的病例并不少见，该文章"胸壁骨性重建的研究进展"比较详细的介绍了胸壁修复与重建的原则和方法，对近几年兴起的3-D打印技术与3-D打印材料植入进行了介绍。临床上胸壁缺损主要见于胸壁肿瘤切除，包括原发胸壁肿瘤，直接侵犯胸壁的肿瘤(如肺癌侵犯胸壁)以及单纯胸壁转移瘤的切除。胸壁缺损按组织来源和部位分为：①软组织缺损；②骨性缺损；③混合缺损(即有软组织缺损又有骨性缺损，如全层胸壁缺损)；④复合缺损(合并有邻近器官组织如颈部、锁骨、膈肌、胸椎和腹壁的缺损)。对于临床诊断胸壁肿瘤而言，手术切除是其主要治疗方法，但术前应做好如下准备：①术前必须明确诊断，排除胸壁结核(初诊)、淋巴瘤和多发性骨髓瘤等非手术治疗疾病；②排除胸壁肿瘤是否远处转移；③对于胸壁巨大肿瘤需进行营养评估，因为创伤大，渗出多，消耗大，白蛋白下降明显且不易纠正，影响伤口愈合；④如胸壁肿瘤破溃，应做细菌培养，排除铜绿杆菌等特殊感染。

骨性胸壁缺损修复与重建的原则尚未完全统一。我们的经验体会是：术前首先要依据胸壁缺损部位、缺损程度、缺损大小和邻近器官组织复合缺损情况综合制定出不同部位和不同方法的胸壁修复与重建。

1.胸壁缺损的部位：不同部位的胸壁缺损，其胸壁修复与重建的侧重点有一定差异。

1) 胸骨缺损：重建胸壁完整性和稳固性的同时，侧重坚固性。单纯胸骨柄缺损可采用Prolene网和转移双侧胸大肌肌瓣或骨性胸壁修复；胸骨体缺损须采用骨性胸壁重建。

2) 上胸壁前、后缺损：因胸壁上部有肩胛骨、胸大肌、胸小肌、乳房(女性)等较厚组织覆盖，对于缺损较小者可直接缝合或局部肌瓣转移或保留其缺损不予修复，但要注意肩胛下角如位于缺损区应做胸壁缺损修复，防止术后肩胛活动产生疼痛；如果缺损较大要进行胸壁缺损修复。

3) 胸壁两侧及胸壁下部缺损：不能直接缝合时均要重建胸壁。

4) 胸腹壁联合缺损(包括胸壁、膈肌和腹壁缺损)：a、部分胸腔腹腔化：如果胸壁缺损上缘在膈肌顶以下，可以将膈肌或膈肌缺损的修复网片与缺损上缘缝合，消除膈肌窦，使其参与腹式呼吸，防止反常呼吸；b、如胸壁缺损超过膈肌顶须进行胸壁重建。

2.胸壁缺损的大小：原则上肺癌侵犯胸壁需切除所侵肋骨的上、下各一正常肋骨，其余肿瘤切除应距肿瘤边缘5 cm，并切缘多点取样送冰冻切片检查。对于胸壁缺损面积 < 5 cm×5 cm者，可采用保留骨性缺损或邻近肌肉、肌瓣缝合填补缺损或网片修补后局部肌瓣、肌皮瓣转移覆盖。而胸壁缺损面积 > 5 cm×5 cm者，建议采用人工材料和自身组织材料重建胸壁。

3.胸壁缺损的程度：包括软组织缺损、骨性缺损、混合缺损(包括全层胸壁缺损)。根据胸壁缺损的大小及程度等做好自身组织材料和人工材料的准备。

目前胸壁缺损修复的方法和材料较多。主要的胸壁缺损修复材料包括自身组织材料和人工材料两大类。常用的自身组织材料如背阔肌肌瓣、背阔肌肌皮瓣、胸大肌肌瓣、胸大肌岛状肌皮瓣、乳房(女性)、腹直肌肌瓣、腹直肌肌皮瓣(transverse rectus abdominis myocutaneous flap, TRAM)、下腹壁穿支皮瓣(deep inferior epigastric perforator flap, DIEP)、膈肌、大网膜股外侧肌皮瓣等。常用的人工材料有钢丝、钢板、肽板、肽网、合成网片、骨水泥、3-D打印肽合金植入材料和3-D虚拟模型、实物模型以及手术导板等辅助手术方法。

目前3-D打印技术和3-D打印材料植入已进行临床应用，如骨科、神经外科等。我科自2014年5月进行3-D打印模型及手术导板应用于胸壁肿瘤切除，2015年9月6日应用3-D打印肽合金材料植入修复和重建胸壁缺损至今，已临床应用12例肽合金材料植入修复胸壁巨大缺损。我们认为3-D打印技术的临床应用流程和原则包括四个部分：①根据肿瘤所采集的放射线数据进行电脑3-D虚拟现实模型(virtual reality, VR)，了解肿瘤的形状、侵犯情况以及与邻近器官组织的关系。在放射线数据采集时，根据肿瘤所产生的来源不同，其采集放射线数据方法也不一样：肿瘤以软组织为主时需做1 mm-2 mm的薄层增强MRI；肿瘤以骨性组织为主时需做1 mm-2 mm的薄层增强CT；②3-D打印胸壁肿瘤实物模型(1:1)，根据胸壁肿瘤情况确定肿瘤切除范围以及手术导板、植入材料模型的设计方案；③3-D打印肿瘤切除范围的实物模型(1:1)及手术导板、植入材料模型，修改和确定手术导板与切除范围是否吻合、植入材料模型是否精准。有时我们采用增强现实(augmented reality, AR)和混合现实技术(mixed reality, MR)替代手术导板；④定型后按植入材料模型数据3-D打印胸壁重建肽合金材料。经过几年的3-D技术的临床应用我们体会3-D打印模型、手术导板和材料置入有如下优点：①3-D打印模型更能使医生直观地了解肿瘤情况和切除范围；②在与病人和家属沟通中变得很容易和易懂；③手术导板的应用能在术中指示手术切除的范围，肿瘤的切除范围更加精准；④3-D打印材料的制作更接近于正常胸廓，对心肺功能的影响减少到最低，且术后外观更接近正常；⑤由于术前已制作胸壁缺损的3-D打印植入材料，大大的缩短了手术时间。总之，3-D打印技术和材料应用于临床一定会得到大力发展。
